# Long-term oral administration of Huaier granules improves survival outcomes in hepatocellular carcinoma patients within Milan criteria following microwave ablation: a propensity score matching and stabilized inverse probability weighting analysis

**DOI:** 10.3389/fphar.2024.1336347

**Published:** 2024-05-07

**Authors:** Kailing Xie, Mingxiu Ma, Feng Xu

**Affiliations:** Department of General Surgery, Shengjing Hospital of China Medical University, Shenyang, China

**Keywords:** complementary alternative medicine, traditional Chinese medicine, vanderbylia robiniophila (Murrill), poria rbiniophila (Murrill) ginns, trametes rbiniophila Murr, Huaier granule, hepatocellular carcinoma, microwave ablation

## Abstract

**Objective:**

This study aimed to elucidate the therapeutic effects of Huaier granules on hepatocellular carcinoma (HCC) within the Milan criteria in patients who underwent microwave ablation (MWA).

**Materials and methods:**

A total of 228 patients were included, with 97 in the Huaier group and 131 in the control group. We evaluated progression-free survival (PFS), overall survival (OS), and extrahepatic metastasis survival (EMS) using Kaplan–Meier (KM) curves with a log-rank test. Propensity Score Matching (PSM) and Stabilized Inverse Probability of Treatment Weighting (IPTW) were performed to minimize selection and confounding biases.

**Results:**

Following PSM, the 1-, 3-, and 5-year PFS rates in the Huaier and control groups were 83.5% vs 70.7%, 57.7% vs 42.6%, and 43.6% vs 31.9% (*p* = 0.030), respectively. The 1-, 3-, and 5-year OS rates were 98.9% vs 95.6%, 83.9% vs 72.3%, and 72.2% vs 53.7% (*p* = 0.023), respectively. The corresponding 1-, 3-, and 5-year EMS rates were 98.9% vs 93.4%, 91.7% vs 83.7%, and 91.7% vs 78.5% (*p* = 0.039), respectively. Stabilized IPTW analysis of KM curves yielded results similar to those of the PSM analysis. Additionally, administering Huaier granules for at least 6 months significantly improved PFS and OS.

**Conclusion:**

Huaier granules can reduce the risk of recurrence and improve the OS of patients with HCC within the Milan criteria following MWA. Administering Huaier granules for over 6 months proved beneficial.

## 1 Introduction

Hepatocellular carcinoma (HCC) stands as one of the most prevalent cancers (Vogel et al., 2022) and ranks as the fourth leading cause of cancer-related mortality globally (Villanueva, 2019; Finn et al., 2020). While surgical interventions like hepatectomy or liver transplantation offer potential cures for HCC patients (Vibert et al., 2020), their applicability and feasibility are limited by factors such as anatomical constraints, inadequate liver function reserve (Lahat et al., 2014), scarcity of liver donors, and the necessity for lifelong immunosuppression (Kulik et al., 2018). In recent years, local thermal ablation techniques, including radiofrequency ablation (RFA) and microwave ablation (MWA), have emerged as viable alternatives for early-stage HCC treatment (Yang et al., 2020). Notably, MWA distinguishes itself with quicker heating over a larger volume, reduced vulnerability to heat sinks, and improved local perfusion compared to RFA (Tan et al., 2019). Despite these advantages, the overall prognosis for early-stage HCC patients undergoing MWA remains poor, with a 5-year recurrence rate reaching approximately 70% (Dong et al., 2023). Although various postoperative adjuvant therapies (AT) have been explored to enhance outcomes for HCC patients (Bruix et al., 2015; Omata et al., 2017; Chen et al., 2018; Heimbach et al., 2018), none have yet been endorsed as adjuvant therapy following MWA.

Botanical drugs play a vital role in the discovery of novel clinical drugs (Liu et al., 2019). *Vanderbylia robiniophila* (Murrill), also known as *Poriar biniophila* (Murrill) Ginns or *Trametes rbinioiophila* Murr, commonly referred to as Huaier, is a sandy beige mushroom found on tree trunks with a historical presence dating back nearly 1,600 years in ancient China (Wang et al., 2023). Similar to many medicinal fungi, Huaier encompasses a complex array of constituents, including polysaccharides, proteins, ketones, alkaloids, and more. Ongoing research on the active components highlights polysaccharides and proteoglycans as the primary bioactive substances. The predominant method for Huaier granules production involves aqueous extraction, yielding a composition containing 41.5% polysaccharide, 12.93% amino acid, and 8.72% water (Sun et al., 2013). The detailed constituents of Huaier extract include six monosaccharides and 18 amino acids (Sun et al., 2013; Qi et al., 2020), as outlined in Supplementary Material S1; Table 1. Additionally, sucrose, dextrin, and soluble starch are added to the aqueous extract of Huaier in a ratio of 2:2:1, forming a granule adjuvant known as Huaier granule.

**TABLE 1:** Baseline Characteristics by treatment group in the total cohort, PSM cohort, and Stabilized IPTW cohort.

Variables	Total cohort			PSM cohort			Stabilized IPTW cohort		
	Huaier group	Control group	*p*-Value	Huaier group	Control group	*p*-Value	Huaier group	Control group	*p*-Value
	*n* = 97	*n* = 131		*n* = 92	*n* = 92		*n* = 96.6	*n* = 130.8	
**Age (years)**	55.98 (9.97)	58.06 (9.15)	0.104	56.2 (9.62)	56.33 (8.13)	0.921	57 (9.96)	57.12 (9.2)	0.931
**Gender**			0.070			0.588			0.938
Male	79 (81.4)	93 (71.0)		74 (80.4)	71 (77.2)		73.4 (75.9)	98.7 (75.4)	
Female	18 (18.6)	38 (29.0)		18 (19.6)	21 (22.8)		23.3 (24.1)	32.1 (24.6)	
**BMI (kg/m** ^**2**^ **)**	23.94 (21.72, 25.61)	23.83 (21.72, 26.28)	0.597	24.09 (21.77, 25.63)	23.75 (22.12, 26.15)	0.678	24.22 (21.77, 25.71)	23.66 (21.61, 26.12)	0.785
**AFP (ng/mL)**			0.582			1.000			0.945
≤400	84 (86.6)	110 (84.0)		79 (85.9)	79 (85.9)		82.6 (85.5)	111.3 (85.1)	
>400	13 (13.4)	21 (16.0)		13 (14.1)	13 (14.1)		14.1 (14.5)	19.5 (14.9)	
**Hepatopathy**			0.512			0.822			1.000
No	4 (4.1)	10 (7.6)		4 (4.3)	6 (6.5)		5.7 (5.9)	8.0 (6.2)	
HBV	83 (85.6)	106 (80.9)		80 (87.0)	76 (82.6)		80.2 (83.0)	108.8 (83.1)	
HCV	7 (7.2)	13 (9.9)		7 (8.3)	8 (8.7)		8.9 (9.2)	11.8 (9.0)	
HBV + HCV	3 (3.1)	2 (1.5)		1 (1.1)	2 (2.2)		1.9 (2.0)	2.2 (1.7)	
**ALB (g/L)**	41.9 (36.3, 44.8)	40.3 (36.25, 43.5)	0.142	42.1 (37.03, 45.02)	40.65 (37.58, 44.3)	0.220	41.5 (36.29, 44.5)	40.3 (35.92, 43.5)	0.241
**TBIL (μ mol/L)**	14.1 (10.3, 21.3)	14.3 (9.3, 19.5)	0.494	13.85 (10.2, 20.1)	13.4 (9.1, 19.45)	0.501	13.95 (10.2, 21.42)	14.4 (9.24, 19.83)	0.548
**ALT (U/L)**	29 (22, 42)	30 (21.5, 50.5)	0.530	29 (21, 42)	32 (23, 53.5)	0.066	29 (21, 42)	30.59 (22, 53.61)	0.197
**AST (U/L)**	29 (22, 42)	31 (22.5, 42)	0.801	28 (22, 42)	33 (24, 46.5)	0.190	28 (21.45, 42)	31 (23, 44.37)	0.494
**PT (s)**	12.6 (11.8, 13.7)	12.2 (11.6, 13.4)	0.567	12.6 (11.8, 13.7)	12.1 (11.6, 13.4)	0.373	12.57 (11.7, 13.7)	12.2 (11.6, 13.4)	0.628
**PLT (10** ^**9**^ **)**	105 (60, 152)	105 (72.5, 144.5)	0.662	106.5 (59.5, 153.25)	102 (72, 143.25)	0.868	105.24 (58.57, 151.6)	102.66 (72, 141.64)	0.768
**Child-Pugh**			0.920			0.694			1.000
A	79 (81.4)	106 (80.9)		75 (81.5)	78 (84.8)		78.5 (81.3)	106.3 (81.3)	
B	18 (18.6)	25 (19.1)		17 (18.5)	14 (15.2)		18.1 (18.7)	24.5 (18.7)	
**Cirrhosis**			0.432			0.844			0.829
No	16 (16.5)	27 (20.6)		16 (17.4)	15 (16.3)		17.3 (17.9)	24.9 (19.0)	
Yes	81 (83.5)	104 (79.4)		76 (82.6)	77 (83.7)		79.3 (82.1)	105.9 (81.0)	
**Portal Hypertension**			0.780			0.760			0.961
No	36 (37.1)	51 (38.9)		35 (38.0)	33 (35.9)		36.4 (37.7)	49.7 (38.0)	
Yes	61 (62.9)	80 (61.1)		57 (62.0)	59 (64.1)		60.2 (62.3)	81.1 (62.0)	
**Number**			0.658			0.704			0.886
Single	80 (82.5)	105 (80.2)		76 (82.6)	74 (80.4)		77.4 (80.1)	105.8 (80.9)	
Multiple	17 (17.5)	26 (19.8)		16 (17.4)	18 (19.6)		19.2 (19.9)	25 (19.1)	
**Tumor size (cm)**	2.3 (1.7, 2.7)	2 (1.75, 2.55)	0.472	2.3 (1.7, 2.7)	2.2 (1.8, 2.8)	0.669	2.3 (1.7, 2.7)	2.1 (1.8, 2.6)	0.600
**Ablation route**			0.909			0.761			0.838
Laparoscopic	37 (38.1)	49 (37.4)		34 (37.0)	36 (39.1)		37.8 (39.1)	49.8 (38.0)	
Percutaneous	60 (61.9)	82 (62.6)		58 (63.0)	56 (60.9)		58.9 (60.9)	81.1 (62.0)	

Abbreviation: PSM, propensity score matching; IPTW, inverse probability of treatment weighting; BMI, body mass index; AFP, alpha-fetoprotein; ALB, albumin; TBIL, total bilirubin; ALT, alanine aminotransferase; AST, aspartate aminotransferase; PT, prothrombin time; PLT, platelet.

Currently, Huaier granules have secured approval in China, either as a standalone therapy or as part of traditional Chinese medicine (TCM) multitherapy, for a spectrum of cancers including gastric cancer (Wang et al., 2019; Hou et al., 2020), lung cancer (Jin et al., 2024), malignant lymphoma, osteosarcoma, leukemia (Wang et al., 2021), breast cancer (Yao et al., 2020), pancreatic adenocarcinoma (Zhou et al., 2020), colorectal cancer (Li B. et al., 2022), and liver cancer (Chen et al., 2018), sanctioned by the Chinese State Food and Drug Administration. Findings from both a multicenter randomized clinical trial and a retrospective study suggest that Huaier granules demonstrate potential in preventing HCC recurrence and extending overall survival (OS) post-resection (Chen et al., 2018; Luo and Hu, 2023). Although another retrospective study reported a significant prevention of early-stage HCC recurrence after thermal ablation with Huaier granules, it did not observe a prolonged OS (Wang et al., 2021). Notably, the study involved two thermal ablation techniques, including RFA and MWA, which may influence tumor recurrence and survival differently. In light of these limitations, our study aims to investigate the therapeutic effects of Huaier granules in HCC within Milan criteria following MWA.

## 2 Materials and method

### 2.1 Study populations

We retrospectively collected data from patients who underwent laparoscopic or ultrasound-guided percutaneous MWA between 2012 and 2020 at the Department of General Surgery, Shengjing Hospital of China Medical University, selected from the electronic medical record database. All patients received a preoperative diagnosis of HCC via contrast ultrasound (US), enhanced multidetector computed tomography (CT), and enhanced magnetic resonance imaging (MRI) scans, accompanied by an alpha-fetoprotein (AFP) test. The diagnostic criteria for HCC are as follows: for lesions with a diameter ≤2 cm, there should be a presence of at least two imaging findings typical of HCC. For lesions with a diameter >2 cm, a minimum of one imaging finding typical of HCC is required. Typical imaging findings include enhanced lesions in the arterial phase (usually late arterial phase), significant strengthening, and a ‘fast in and fast out’ enhancement pattern, with a decrease in the portal vein and/or delayed phase. The inclusion criteria were as follows: (a) diagnosis of HCC; (b) within the Milan criteria: i) single tumor lesion ≤5 cm in diameter or 2-3 tumor nodules with a maximum diameter ≤3 cm. ii) No invasion of vessels or bile ducts. iii) Absence of lymph node or extrahepatic metastasis. (c) Complete ablation, defined as a postoperative imaging test showing an ablation zone with a margin (≥5 mm) covering the original tumor size and devoid of any HCC features. The exclusion criteria were as follows: (a) patients with cardiovascular disease or immune system disorders; (b) Child-Pugh score C; (c) repeated carcinoma; (d) open abdominal ablation; (e) hepatectomy combined with ablation; (f) other preoperative treatments; (g) postoperative anti-tumor drug or other Chinese medicine therapy; (h) abnormal renal function.

Demographic characteristics and clinical features, including sex, age, body mass index (BMI), alpha-fetoprotein (AFP), hepatopathy, platelet (PLT), albumin (ALB), total bilirubin (TBIL), alanine aminotransferase (ALT), aspartate aminotransferase (AST), prothrombin time (PT), Child-Pugh score, tumor size, tumor number, presence of cirrhosis, and presence of hypertension, were collected. The study adhered to the ethical guidelines of the Declaration of Helsinki and received approval from the Ethics Committee of Shengjing Hospital of China Medical University. Written informed consent was obtained from all the patients or their representatives for enrollment in this study. To protect patient privacy, we de-identified all data containing personal information, such as names, hospital IDs, and telephone numbers.

### 2.2 MWA procedure

All patients underwent US-guided percutaneous or laparoscopic MWA using a specialized cooled shaft system (ECO-100AI10, ECO Microwave System Co., Nanjing, China) with a maximum power of 80 W at 2,450 MHz in our institution. The system is equipped with real-time temperature monitoring and cooling circulation technology. The MWA procedures were performed by experienced hepatobiliary surgeons with 5–10 years of expertise in MWA. Prior to treatment, a thorough US examination was conducted for precise tumor localization and strategic treatment planning. Patients were given the option of percutaneous MWA or laparoscopic MWA based on tumor localization and their specific needs. The placement of the antenna during the procedure varied based on tumor diameter: for tumors with a 2 cm diameter, the antenna was centrally placed on the tumor. Tumors ranging from 2 to 3 cm in diameter had antennas placed on both sides. Tumors exceeding 3 cm underwent multiple overlapping ablations achieved by repositioning the antenna. The sequential placement on different areas of the tumor was tailored according to its size and shape. The surgical objective was to achieve complete tumor ablation, ensuring a margin exceeding 1 cm for each ablation. This meticulous approach aimed to optimize the effectiveness of the MWA treatment.

### 2.3 Taxonomy, complete species name, and synonymy of Huaier

Based on Index Fungorum (https://www.indexfungorum.org/names/NamesRecord.asp?RecordID=826677), Species Fungorum current name:

Vanderbylia robiniophila (Murrill) B.K. Cui and Y.C. Dai, in Cui, Li, Ji, Zhou, Song, Si, Yang & Dai 2019.

Basionym:

*Trametes robiniophila* Murrill 1907.

Position in classification:

Polyporaceae, Polyporales, Incertae sedis, Agaricomycetes, Agaricomycotina, Basidiomycota, Fungi.

Species Fungorum synonymy:

*Trametes robiniophila* Murrill, N. Amer. Fl (New York) 9(1): 42 (1907).

*Polyporus robiniophilus* (Murrill) Lloyd, Mycol. Writ (Cincinnati) 4(Letter 42): 12 (1912).

*Perenniporia robiniophila* (Murrill) Ryvarden, Mycotaxon 17: 517 (1983).

*Poria robiniophila* (Murrill) Ginns, Mycotaxon 21: 331 (1984).

### 2.4 Production of Huaier granules

Sucrose, dextrin, and soluble starch were meticulously combined with the aqueous extract of Huaier in a precise ratio of 2:2:1, resulting in the creation of a granule adjuvant known as Huaier granule. Produced by Jiangsu Qidong Gaitianli Pharmaceutical Co., Ltd., China, the recommended dosage was 20 g, administered three times a day. In the manufacturing process of Huaier granules, chromatographic fingerprint analysis played a pivotal role in illustrating the “plant equivalence” of the product concerning raw material preparation and quality control. This analysis ensured that the active constituents met the stringent requirements set forth by the State Food and Drug Administration of China (SFDA). From March 1993 to June 1994, a phase III clinical trial of Huaier Granules was conducted, holding the production certificate no. Z20000109. Notably, Huaier Granules received the first new drug certificate issued by the SFDA in 2002. Each production lot adhered to the Good Manufacturing Practice standards, complying with the quality assurance standards mandated by the SFDA (identification code: WS3-215 (Z-029)-2001(Z)-2012; ybz042020032009z-2012). Detailed fingerprints are provided in Supplementary Material S1.

### 2.5 Follow-up

Patients underwent re-examinations at the outpatient department of Shengjing Hospital, the inpatient department of Shengjing Hospital, or their local hospital 2 months after surgery. These follow-ups included US, CT, MRI, and monitoring of AFP levels. Subsequent examinations occurred every 3 months. Prognostic data and the cumulative use of Huaier granules were collected from the electronic medical record database or through follow-up appointments. Patients made decisions regarding the intake of Huaier granules based on their preferences and economic status at the time of discharge. Patients were categorized into Huaier and control groups based on the cumulative use of Huaier granules for more than 3 months during the follow-up period. For patients experiencing a relapse, treatment options were determined based on factors such as the number, size, and location of the tumor, as well as the patient’s liver function status. Progression**-**free survival (PFS) was defined as the duration from the end of the initial surgery to the earliest detection of tumor recurrence or the date of the last follow-up. OS was defined as the time elapsed from the end of the initial surgery to either the date of death or the last follow-up. Extrahepatic metastasis survival (EMS) referred to the span from the end of the initial surgery to the initial identification of extrahepatic tumor metastasis or the date of the last follow-up.

### 2.6 Propensity score matching and stabilized inverse probability of treatment weighting

To mitigate selection and confounding biases in our study, we employed propensity score matching (PSM) and stabilized inverse probability of treatment weighting (IPTW) (Reiffel, 2020). The propensity score (PS) was calculated using logistic regression, incorporating the following clinical features: sex, age, BMI, AFP, hepatopathy, Child-Pugh score, Cirrhosis, PLT, ALT, AST, presence of hypertension, presence of multiple tumors, and tumor size.

PSM was performed using a 1:1 ratio employing the nearest neighbor matching algorithm with an optimal caliper of 0.2 for both the Huaier and control groups. For Stabilized IPTW, the weighting coefficients for patients in the Huaier and control groups were PT/PS and (1 − PT)/(1 − PS) (PT = patients in the Huaier group/all patients), respectively. Details regarding the PSM and Stabilized IPTW analysis can be found in Supplementary Material S2. These methods were crucial in ensuring robustness and reliability in addressing biases during the evaluation of treatment outcomes.

### 2.7 Other statistical analyses

For continuous variables, mean ± standard deviation was used if they conformed to the normal distribution; otherwise, they were presented as median (quartile 25%, 75%). The comparison between the two groups for continuous data involved the *t*-test for normally distributed data and a non-parametric test for non-normally distributed data. Categorical variables were compared using Fisher’s exact test or the chi-square test. PFS, OS, and EMS were assessed using the Kaplan-Meier method with a log-rank test. The association between clinicopathological variables and PFS, OS, and EMS was assessed using univariate Cox proportional hazards regression analysis. Multivariate Cox proportional hazard regression analyses were performed to assess the relationship between Huaier granules and prognostic outcomes. R version 4.1.2 and SPSS version 26.0 (SPSS, Chicago, IL) were used for statistical analyses, with a significance level set at *p* < 0.05.

## 3 Results

### 3.1 Baseline characteristics

**FIGURE 1:**
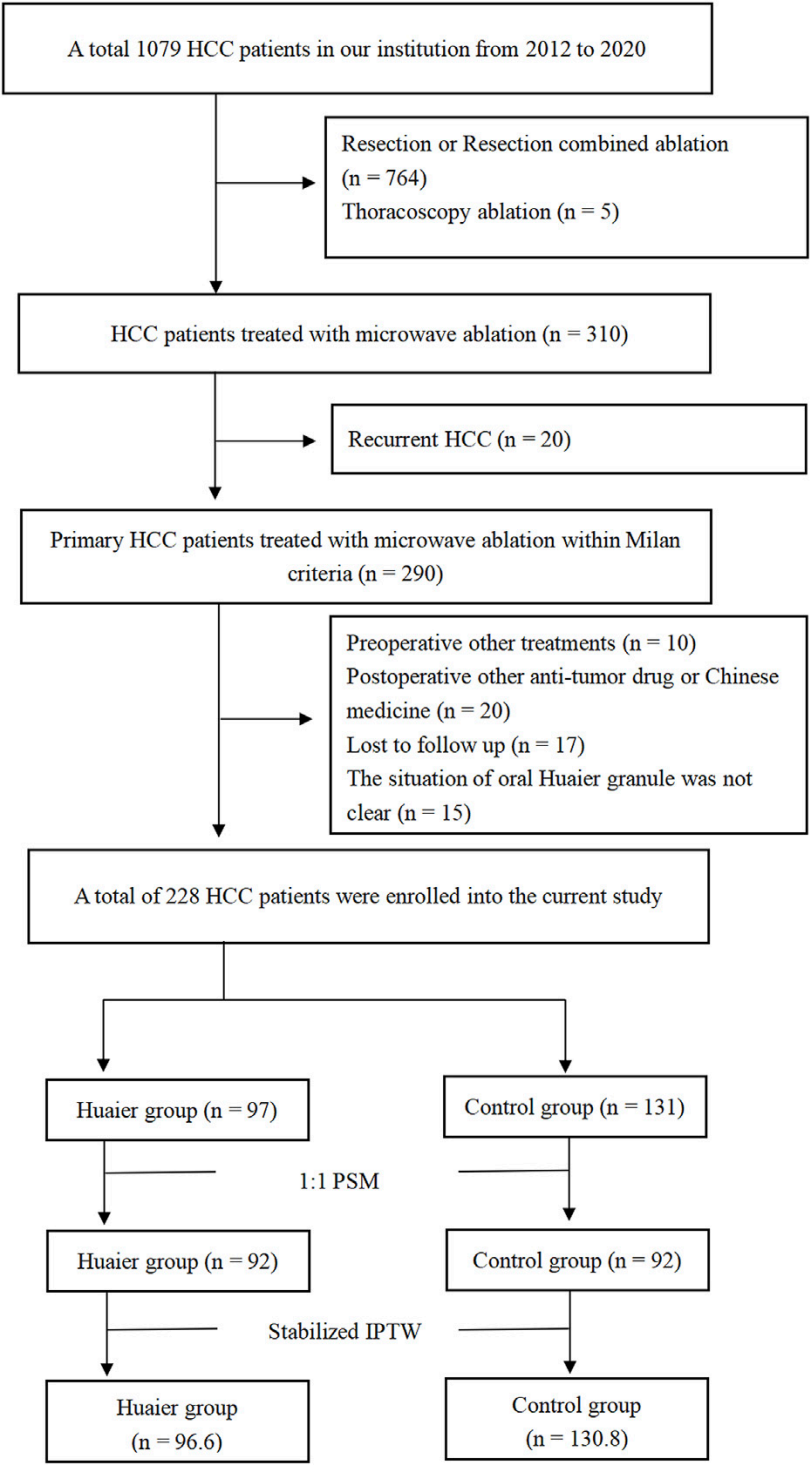
Flowchart of patient selection.

A total of 228 patients (172 males and 56 females; age range: 32–80 years) were enrolled in this study (Figure 1). The patients were divided into the Huaier (n = 97) and control (n = 131) groups. After PSM, the Huaier and control groups were matched with 92 patients. After Stabilized IPTW, there were 96.6 patients in the Huaier group and 130.8 patients in the control group, respectively. The baseline clinical characteristics of the Huaier and control groups are listed in Table 1. No significant differences were found in Age, Sex, BMI, AFP, Hepatopathy, ALB, TBIL, ALT, AST, PT, PLT, Child-Pugh score, Cirrhosis, the presence of cirrhosis, the presence of hypertension, the presence of multiple tumors, tumor size, ablation route, whether in total cohort, in the PSM cohort, or in the Stabilized IPTW cohort (Table 1).

In the Huaier group, the median follow-up time was 42 months (ranged 4–105). During the follow-up period, 19 (19.6%) patients died, and 43 (44.3%) patients developed tumor progression, including 10 (10.3%) patients with local recurrence, 27 (27.8%) patients with intrahepatic recurrence, and 6 (6.2%) patients with extrahepatic recurrence. In the control group, the median follow-up time was 47 months (ranged 4–104), 44 (33.6%) patients died, and 79 (60.3%) patients developed tumor progression, including 17 (13.0%) patients with local recurrence, 40 (30.5%) patients with intrahepatic recurrence, and 22 (16.8%) patients with extrahepatic recurrence.

### 3.2 Comparison of PFS, OS, and EMS between the Huaier group and the control group

**FIGURE 2:**
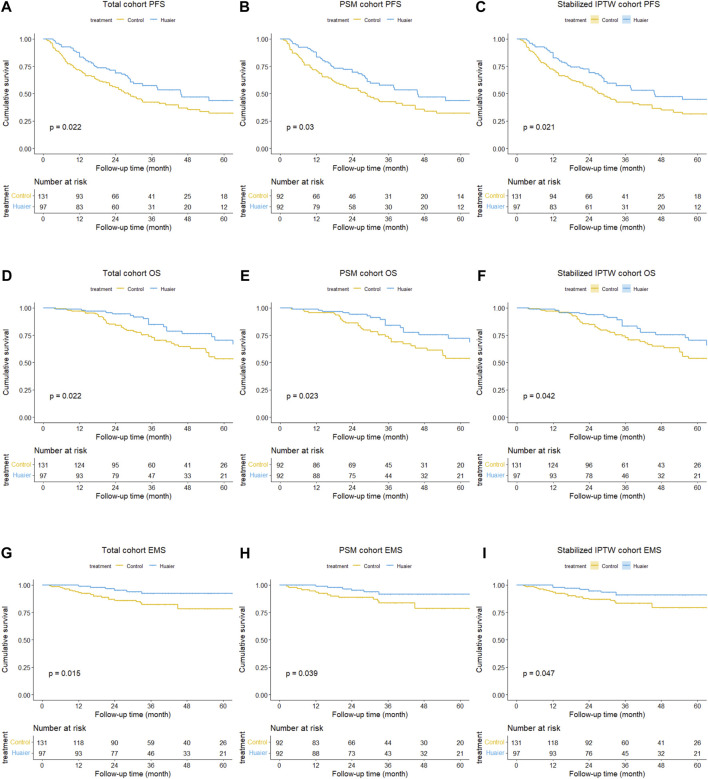
Differences in progression**-**free survival between the Huaier and control groups in the total cohort **(A)**, PSM cohort **(B)**, and Stabilized IPTW cohort **(C)**. Similarly, differences in overall survival between the Huaier and control groups in the total cohort **(D)**, PSM cohort **(E)**, and Stabilized IPTW cohort **(F)**. Additionally, differences in extrahepatic metastasis survival between the Huaier and control groups in the total cohort **(G)**, PSM cohort **(H)**, and Stabilized IPTW cohort **(I)**.

Before PSM or Stabilized IPTW, the cumulative 1-, 3-, and 5-year PFS rates in the Huaier and control groups were 83.3% vs*.* 70.9%, 57.5% vs*.* 42.1%, and 43.6% vs*.* 32.0%, respectively (*p* = 0.022, Figure 2A). The 1-, 3-, and 5-year OS rates were 99.0% vs*.* 96.9%, 84.7% vs*.* 72.8%, and 70.3% vs*.* 53.3%, respectively (*p* = 0.022; Figure 2D). The corresponding 1-, 3-, and 5-year EMS rates were 98.9% vs*.* 93.0%, 92.2% vs*.* 82.3%, and 92.2% vs*.* 78.4% (*p* = 0.022, Figure 2G), respectively.

After PSM, the cumulative 1-, 3-, and 5-year PFS rates in the Huaier and control groups were 83.5% vs*.* 70.7%, 57.7% vs*.* 42.6%, and 43.6% vs*.* 31.9% (*p* = 0.030, Figure 2B), respectively. The 1-, 3-, and 5-year OS rates were 98.9% vs*.* 95.6%, 83.9% vs*.* 72.3%, and 72.2% vs*.* 53.7%, respectively (*p* = 0.023; Figure 2E). The corresponding 1-, 3-, and 5-year EMS rates were 98.9% vs*.* 93.4%, 91.7% vs*.* 83.7%, and 91.7% vs*.* 78.5% (*p* = 0.039, Figure 2H), respectively.

Following Stabilized IPTW, the cumulative 1-, 3-, and 5-year PFS rates in the Huaier and control groups were 82.7% vs*.* 71.5%, 57.2% vs*.* 42.1%, and 44.8% vs*.* 31.5%, respectively (*p* = 0.021, Figure 2C). The corresponding 1-, 3-, and 5-year OS rates were 98.9% vs*.* 97.2%, 83.5% vs*.* 73.0%, and 66.8% vs*.* 53.8%, respectively (*p* = 0.042, Figure 2F). The corresponding 1-, 3-, and 5-year EMS rates were 97.8% vs*.* 93.3%, 91.0% vs*.* 83.2%, and 91.0% vs*.* 79.3%, respectively (*p* = 0.047, Figure 2I).

### 3.3 Association between Huaier granules and prognostic outcomes based on multivariate Cox regression analysis

The results of the univariate Cox regression analysis are presented in Supplementary Material S3; Table 1. Three models were established to assess the relationship between Huaier granules and prognostic outcomes with adjusting factors (*p* < 0.1, univariate analysis), and Huaier granules were found to be associated with better PFS [HR 95CI% 0.56 (0.38–0.82), *p* = 0.003], OS [HR 95CI% 0.55 (0.31–0.95), *p* = 0.032], and EMS [HR 95%CI 0.40 (0.16–0.99), *p* = 0.049] in the total cohort, as detailed in Table 2.

**TABLE 2:** Multivariate Cox regression analysis of PFS, OS, and EMS in the total cohort.

PFS			
Crude model		Model 1	
HR (95CI%)	P	HR (95CI%)	P
0.65 (0.45–0.94)	0.024	0.56 (0.38–0.82)	0.003

OS			
Crude model		Model 2	
HR (95CI%)	P	HR (95CI%)	P
0.54 (0.31–0.92)	0.024	0.55 (0.31–0.95)	0.032

EMS			
Crude model		Model 3	
HR (95CI%)	P	HR (95CI%)	P
0.34 (0.14–0.84)	0.020	0.40 (0.16–0.99)	0.049

Model 1: Huaier adjusted for Gender, AFP, grade; TBIL, grade, size grade, and ablation route.

Model 2: Huaier adjusted for Age, Hepatic virus, and ALB, grade. Model 3: Huaier adjusted for Age, ALT, grade, and ablation route.

Abbreviation: PFS, Progression-free survival; OS, overall survival; EMS, extrahepatic metastasis survival; AFP, alpha-fetoprotein; TBIL, total bilirubin; ALB, albumin; ALT, alanine aminotransferase.

### 3.4 Therapeutic effect of cumulative administration of Huaier granules on prognostic outcomes

**FIGURE 3:**
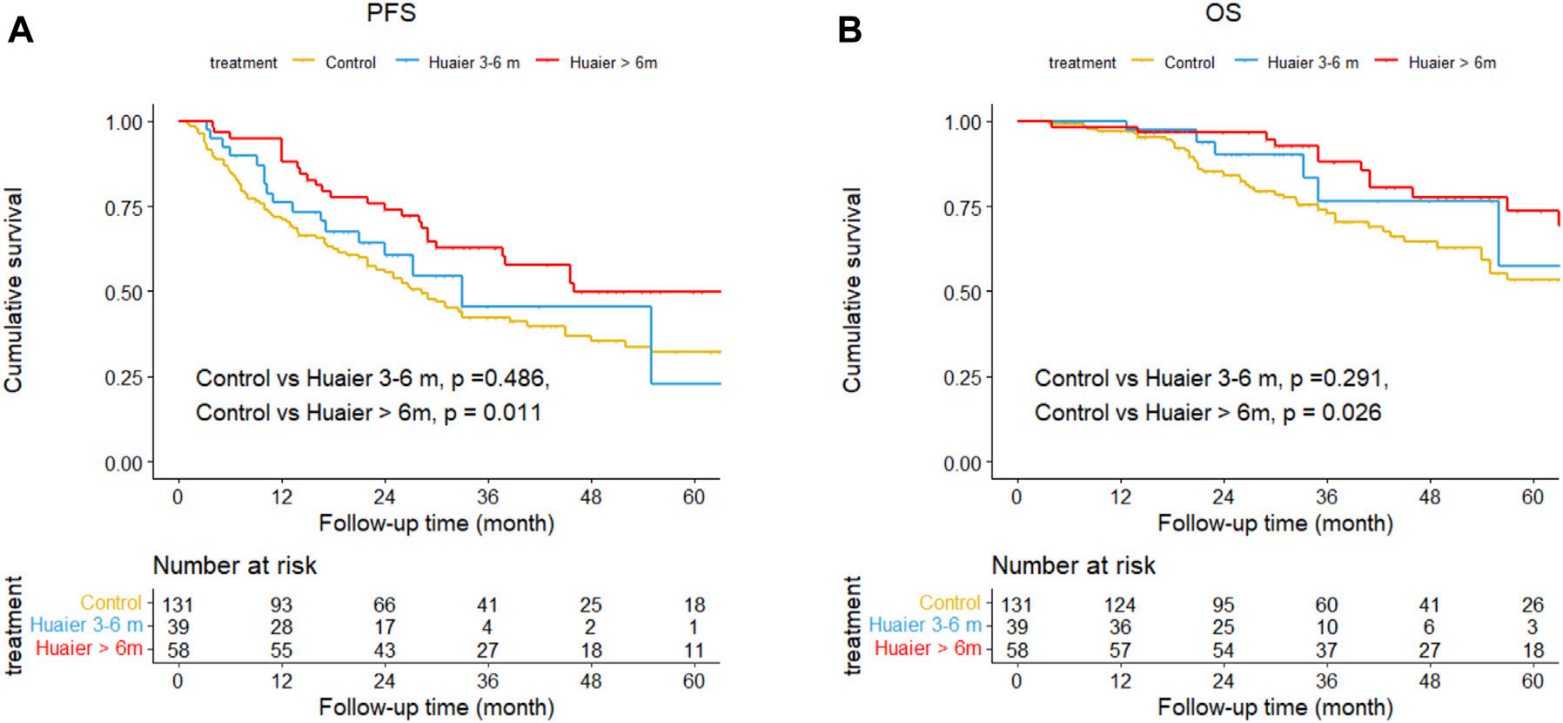
Differences in progression**-**free survival **(A)** and overall survival **(B)** among the Huaier >6 months group, Huaier 3–6 months group, and Control group in the total cohort.

The cumulative 1-, 3-, and 5-year PFS rates among the Huaier >6 months, Huaier 3–6 months and control groups were 87.9% vs. 76.1% vs*.* 70.9%, 62.6% vs. 45.3% vs*.* 42.1%, and 49.7% vs. 49.7% vs*.* 22.7% (Huaier >6 months vs*.* control, *p* = 0.011; Huaier 3–6 months vs*.* control, *p* = 0.486; Figure 3A). The cumulative 1-, 3-, and 5-year OS rates among the Huaier >6 months, Huaier 3–6 months, and control groups were 98.3% vs. 100.0% vs. 96.9%, 87.9% vs. 76.4% vs*.* 72.8%, and 73.7% vs. 57.3% vs*.* 53.3% (Huaier >6 months vs*.* control, *p* = 0.026; Huaier 3–6 months vs*.* control, *p* = 0.291; Figure 3B).

We established two models with adjusting factors (*p* < 0.1 in the univariate analysis), the results showed Huaier >6 months was associated with better PFS [HR 95CI% 0.47 (0.30–0.75), *p* = 0.001] and OS [HR 95CI% 0.51 (0.27–0.97), *p* = 0.040], but Huaier 3–6 months was not [PFS, HR 95CI% 0.78 (0.46–1.33), *p* = 0.365; OS, HR 95CI% 0.63 (0.27–1.49), *p* = 0.295, Table 3]. Although the Huaier 3–6 months group was not associated with worse PFS [HR 95CI% 1.65 (0.46–1.33), *p* = 0.114; Supplementary Material S3; Table 2] and OS than Huaier > 6 m [HR 95CI% 1.21 (0.46–3.21), *p* = 0.704; Supplementary Material S3; Table 2], there was a significant linear trend between the duration of Huaier granules administration and prognostic outcomes (PFS, *p* for trend = 0.001; OS, *p* for trend = 0.032; Table 3 and Supplementary Material S3; Table 2).

**TABLE 3:** Multivariate Cox regression analysis of the therapeutic effect of oral administration duration of Huaier granules on prognostic outcomes.

PFS						
	Crude model			Model 1		
	HR (95CI%)	P	P for trend	HR (95CI%)	P	P for trend
Control	-	-	0.012	-	-	0.001
Huaier 3–6 m	0.85 (0.50–1.43)	0.533		0.78 (0.46–1.33)	0.365	
Huaier >6 m	0.57 (0.36–0.88)	0.012		0.47 (0.30–0.75)	0.001	

OS						
	Crude model			Model 2		
	HR (95CI%)	P	P for trend	HR (95CI%)	P	P for trend
Control	-	-	0.023	-	-	0.032
Huaier 3–6 m	0.65 (0.28–1.53)	0.321		0.63 (0.27–1.49)	0.044	
Huaier >6 m	0.50 (0.27–0.93)	0.028		0.51 (0.27–0.97)	0.040	

Model 1: Huaier adjusted for Gender, AFP, grade; TBIL, grade, size grade, and ablation route.

Model 2: Huaier adjusted for Age, Hepatic virus, and ALB, grade.

Abbreviation: PFS, Progression-free survival; OS, overall survival; AFP, alpha-fetoprotein; TBIL, total bilirubin; ALB, albumin.

### 3.5 Subgroup analysis in the total cohort

**FIGURE 4:**
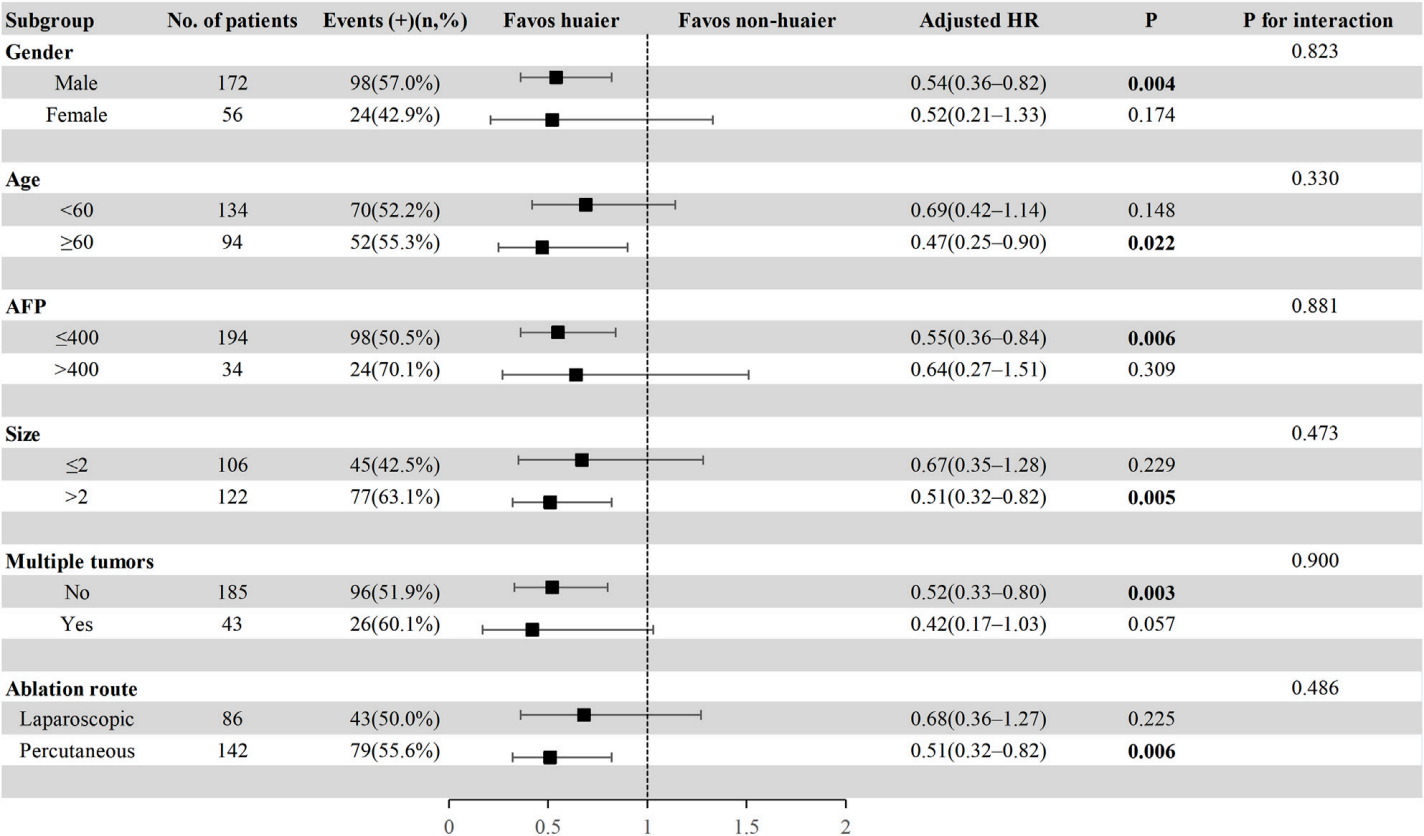
Subgroup analysis of progression**-**free survival in the total cohort based on multivariate Cox regression.

**FIGURE 5:**
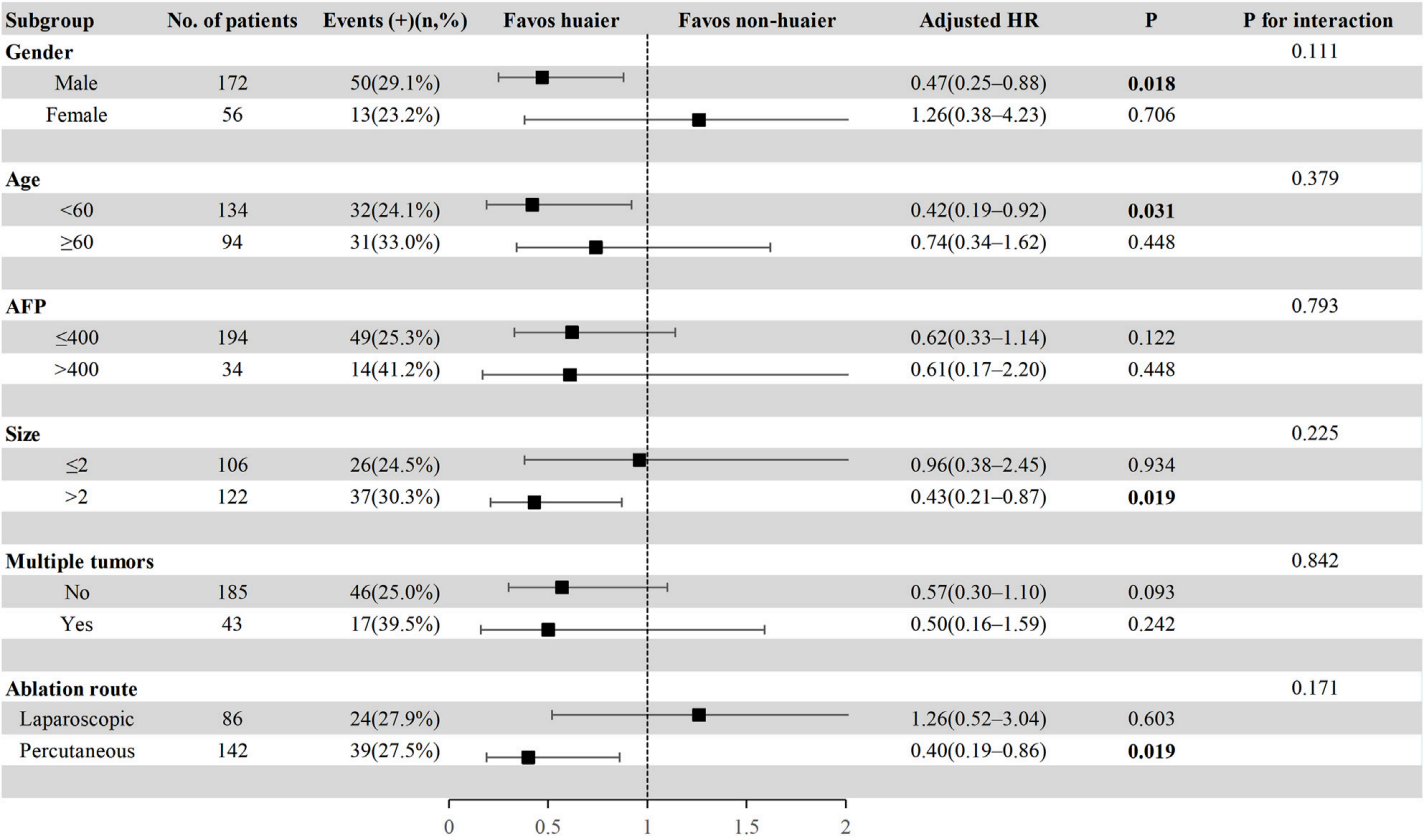
Subgroup analysis of overall survival in the total cohort based on multivariate Cox regression.

To further explore the effect of Huaier granules therapy on prognosis outcomes, including PFS and OS, we performed a multivariate Cox regression stratified analysis across various subgroups within the study population. Subgroup categorizations were based on age (<60 or ≥60 years), gender, AFP levels (≤400 or >400 ng/mL), tumor size (≤2 or >2 cm), presence of multiple tumors (no or yes), and the ablation route (laparoscopic or percutaneous). The results demonstrated a significant association between Huaier granules and a reduced risk of recurrence in specific subgroups, including male, age ≥60 years, AFP ≤400 ng/mL, tumor size >2 cm, single tumor, and percutaneous MWA (Figure 4). Furthermore, Huaier granules were associated with decreased mortality risk in specific subgroups, such as male, age <60 years, tumor size >2 cm, and percutaneous MWA (Figure 5). Notably, no subgroup exhibited a significant interaction with the administration of Huaier granules, affecting neither recurrence nor mortality outcomes. Therefore, we deemed it appropriate to reject the findings of the subgroup analyses.

### 3.6 Treatment-related side effects

In the Huaier group, 25 (25.8%) patients reported treatment-related side effects. These included poor appetite (4.1% of patients), diarrhea (2.1%), nausea (4.1%), emesis (3.1%), dyspepsia (5.2%), fatigue (1%), and fever (6.2%). Conversely, in the control group, 29 (22.1%) patients reported treatment-related side effects, including poor appetite (5.3%), diarrhea (1.5%), naus43ea (4.6%), emesis (2.3%), dyspepsia (5.3%), fatigue (<1%), and fever (2.3%). Notably, there were no significant differences observed in the overall incidence of treatment-related side effects between the Huaier group and the control group (*p* = 0.904) (Table 4).

**TABLE 4:** Comparison of treatment-related side effects in the Huaier and control group.

	Huaier group (*n* = 97)	Control group (*n* = 131)	P
**Theraputic-related effects**	25 (25.8%)	29 (22.1%)	0.904
Poor appetite	4 (4.1%)	7 (5.3%)	
Diarrhea	2 (2.1%)	2 (1.5%)	
Nausea	4 (4.1%)	6 (4.6%)	
Emesis	3 (3.1%)	3 (2.3%)	
Dyspepsia	5 (5.2%)	7 (5.3%)	
Fatigue	1 (1.0%)	1 (<1%)	
Fever	6 (6.2%)	3 (2.3%)	

Therapeutic-related side effects includes the ablation syndrome (usually occurs 1–5 days after MWA) and drug-related side effects (usually occurs 2 weeks after MWA).

## 4 Discussion

The overall prognosis for early-stage HCC treated with MWA is commonly unfavorable (Dong et al., 2023). To improve outcomes in this patient population, adjuvant therapy becomes crucial. However, the prevalence of hepatic reserve dysfunction and cirrhosis among these patients adds complexity to treatment decisions (Aoki et al., 2020). The necessity for an adequate and safe ablation zone inevitably results in liver impairment. Therefore, selecting an adjuvant therapy with minimal impact on liver function is paramount.

The integration of traditional Chinese medicine, drawing on three thousand years of Chinese medical practice, has become a notable approach (Liu et al., 2019; Changou et al., 2021; Li K. et al., 2022). Huaier granules, classified as proprietary Chinese medicines, offer the advantages of low toxicity and convenient administration, demonstrating effectiveness in the comprehensive treatment of HCC (Lei et al., 2015; Chen et al., 2018; Zhou et al., 2018; Wang et al., 2021). A prior study indicated that Huaier granules extended PFS in early-stage HCC following thermal ablation (Wang et al., 2021). However, it is crucial to acknowledge the limitations of the aforementioned study. Firstly, its cohort nature introduced unadjusted selection bias. Despite attempts to address confounding bias through Cox multifactor regression, the results, even after adjusting for all risk factors, indicated no significant association between Huaier granules and improved PFS. Secondly, Huaier granules did not demonstrate a positive impact on OS, necessitating validation in further studies. Lastly, the study did not encompass patients with HCC undergoing laparoscopic MWA. These limitations underscore the need for additional research to provide a more comprehensive understanding of the potential benefits and drawbacks of Huaier granules in the adjuvant treatment of early-stage HCC.

Using PSM and Stabilized IPTW to calibrate the selection confounders, our study revealed significant differences in PFS, OS, and EMS between the Huaier and control groups (Reiffel, 2020). In Cox multivariate regression analyses, the results indicated that Huaier granules were associated with improved PFS, OS, and EMS after adjusting for all risk factors in the models. These findings affirm that Huaier granules can effectively prevent HCC recurrence within the Milan criteria following MWA, aligning with prior research. However, the ongoing controversy regarding whether Huaier granules enhance OS persists, necessitating further validation studies (Wang et al., 2021). Additionally, despite earlier evidence suggesting a positive linear dose-response relationship between the duration of Huaier granules administration and favorable outcomes, extended use may incur high costs and potential liver damage. Therefore, determining the minimum effective duration for Huaier granules becomes crucial. Our study discovered that administering Huaier granules for at least 6 months significantly improved prognosis. Regrettably, subgroup analyses suggested potential benefits for certain population subgroups, but the non-significant results of the interaction analyses indicated the unreliability of these subgroup analyses.

Numerous laboratory experiments have unveiled the potential mechanism behind Huaier’s anti-tumor therapy (Zhu et al., 2022; Luo et al., 2023; Jin et al., 2024; Li et al., 2024). This includes the downregulation of cyclin-dependent kinase, Cyclin D, β-catenin, and other cell cycle regulatory proteins, inducing G1/S phase arrest in HCC cells (Zhang et al., 2015; Bao et al., 2016; Hu et al., 2016; Niu et al., 2020; Xu et al., 2020). Additionally, Huaier has demonstrated the activation of the caspase cascade, leading to HCC cell apoptosis (Zhang et al., 2015; Bao et al., 2016; Xu et al., 2020). It further inhibits epithelial-mesenchymal transition, impeding HCC cell invasion and metastasis (Zheng et al., 2014; Li et al., 2015a). Moreover, Huaier enhances the anti-tumor activity of macrophages by promoting M1 activation and inhibiting macrophage polarization to M2 (Sun et al., 2013; Li et al., 2016; Yang et al., 2019; Zhang et al., 2020). Furthermore, it corrects immune disorders caused by tumor cells (Li et al., 2015b). These mechanisms provide a theoretical foundation for improving HCC prognosis through adjuvant therapy using Huaier granules (Li et al., 2020; Meng et al., 2020).

Our study has several limitations. Firstly, being a single-center retrospective study, despite employing PSM and Stabilized IPTW to mitigate confounding factors, eliminating interference entirely remains challenging. Secondly, the sample size imbalances among subgroups may compromise statistical efficiency for subgroup analyses, potentially yielding false-positive and false-negative results. Finally, we did not explore the impact of Huaier granules on postoperative tumor markers and related immune indexes. Therefore, future clinical trials should incorporate prospective and pre-designed subgroup protocols for a more comprehensive assessment.

In conclusion, our findings suggest that Huaier granules can significantly reduce the risk of recurrence and improve the OS of patients with HCC within the Milan criteria following MWA. Notably, the administration of Huaier granules for over 6 months demonstrated substantial benefits. While acknowledging these positive outcomes, the study’s limitations underline the need for cautious interpretation, and prospective trials with robust designs are warranted to further validate these findings.

## Data Availability

The original contributions presented in the study are included in the article/Supplementary Material, further inquiries can be directed to the corresponding author.
