# Supplementary material for: Long-term oral administration of Huaier granules improves survival outcomes in hepatocellular carcinoma patients within Milan criteria following microwave ablation: a propensity score matching and stabilized inverse probability weighting analysis
Source: Front Pharmacol. 2024 May 7;15:1336347. doi: 10.3389/fphar.2024.1336347 (PMC11106438; doi:10.3389/fphar.2024.1336347)
Supplement: Supplementary file 2 [file Table1.docx]

**Supplementary File 3**

**“Long-Term Oral Administration of Huaier Granules Improves Survival Outcomes in Hepatocellular Carcinoma Patients Within Milan Criteria following Microwave Ablation: A Propensity Score Matching and Stabilized Inverse Probability Weighting Analysis”**

Table 1 Univariate analysis of PFS, OS and EMS in total cohort.

|  | PFS |  |  | OS |  |  | EMS |  |
| --- | --- | --- | --- | --- | --- | --- | --- | --- |
|  | Univariate |  |  | Univariate |  |  | Univariate |  |
|  | HR (95CI%) | P |  | HR (95CI%) | P |  | HR (95CI%) | P |
| Age (years) | 1.01(0.99–1.03) | 0.492 |  | 1.02(0.99–1.05) | 0.084 |  | 1.05(1.01–1.10) | **0.012** |
| Gender (Female) | 0.65(0.42–1.02) | 0.063 |  | 0.81(0.44–1.49) | 0.494 |  | 1.55(0.70–3.42) | 0.281 |
| BMI (>24 Kg/m^2^) | 1.04(0.73–1.48) | 0.842 |  | 1.26(0.76–2.06) | 0.369 |  | 1.31(0.62–2.77) | 0.477 |
| AFP (>400 ng/ml) | 1.58(1.01–2.47) | **0.045** |  | 1.61(0.89–2.91) | 0.117 |  | 1.26(0.48–3.31) | 0.641 |
| Hepatic virus (Yes) | 0.77(0.36–1.66) | 0.505 |  | 0.26(0.12–0.61) | **0.001** |  | NA | 0.997 |
| ALB (>35 g/L) | 0.82(0.54–1.26) | 0.370 |  | 0.56(0.33–0.96) | **0.036** |  | 2.14(0.65–7.09) | 0.213 |
| TBIL (>17.1μmol/L) | 1.38(0.96–1.98) | 0.078 |  | 1.30(0.78–2.16) | 0.310 |  | 1.43(0.67–3.01) | 0.354 |
| ALT (> 40 U/L) | 1.23(0.86–1.78) | 0.258 |  | 1.10(0.66–1.83) | 0.711 |  | 1.91(0.91–4.00) | 0.088 |
| AST (>40 U/L) | 1.11(0.75–1.65) | 0.590 |  | 1.37(0.81–2.32) | 0.239 |  | 1.80(0.84–3.84) | 0.129 |
| PLT (> 100*10^9^) | 1.17(0.82–1.67) | 0.391 |  | 1.28(0.78–2.11) | 0.334 |  | 1.66(077–3.60) | 0.199 |
| ALP (>75 U/L) | 1.17(0.82–1.69) | 0.388 |  | 0.75(0.46–1.23) | 0.259 |  | 1.56(0.70–3.44) | 0.274 |
| GGT (> 50 U/L) | 1.31(0.92–1.87) | 0.136 |  | 1.80(1.10–2.96) | **0.020** |  | 1.49(0.71–3.13) | 0.291 |
| Child-Pugh (B) | 0.99(0.63–1.54) | 0.950 |  | 0.66(0.33–1.34) | 0.253 |  | 0.47(0.14–1.55) | 0.216 |
| Cirrhosis (Yes) | 0.97(0.62–1.53) | 0.900 |  | 0.65(0.36–1.17) | 0.152 |  | 1.31(0.45–3.77) | 0.622 |
| Portal hypertension (Yes) | 0.92(0.64–1.33) | 0.669 |  | 1.11(0.67–1.86) | 0.684 |  | 0.61(0.29–1.29) | 0.195 |
| Multiple (Yes) | 1.34(0.87–2.07) | 0.185 |  | 1.74(1.00–3.04) | 0.052 |  | 1.00(0.38–2.64) | 0.994 |
| Size grade (>2cm) | 1.64(1.14–2.38) | **0.008** |  | 1.19(0.72–1.97) | 0.490 |  | 1.34(0.63–2.86) | 0.451 |
| Ablation route (percutaneous) | 1.38(0.95–2.01) | 0.089 |  | 1.29(0.77–2.15) | 0.331 |  | 1.42(0.98–2.07) | 0.065 |

Abbreviation: BMI, body mass index; AFP, alpha-fetoprotein; ALB, albumin; TBIL, total bilirubin; ALT, alanine aminotransferase; AST, aspartate aminotransferase; PT, prothrombin time; PLT, platelet.

Table 2 Therapeutic effect of oral administration duration of Huaier granule on prognostic outcome based on multivariate analysis.

| **PFS** | | | | | | | |
| --- | --- | --- | --- | --- | --- | --- | --- |
|  | Crude model |  |  |  | Model 1 |  |  |
|  | HR (95CI%) | P | P for trend |  | HR (95CI%) | P | P for trend |
| Huaier > 6 m | - | - | 0.012 |  | - | - | 0.001 |
| Control | 1.76(1.13–2.75) | 0.012 |  |  | 2.11(1.34–3.33) | 0.001 |  |
| Huaier 3-6 m | 1.49(0.81–2.76) | 0.204 |  |  | 1.65(0.46–1.33) | 0.114 |  |
| **OS** | | | | | | | |
|  | Crude model |  |  |  | Model 2 |  |  |
|  | HR (95CI%) | P | P for trend |  | HR (95CI%) | P | P for trend |
| Huaier > 6 m | - | - | 0.023 |  | - | - | 0.032 |
| Control | 2.00(1.08–3.72) | 0.028 |  |  | 1.93(1.02–3.65) | 0.044 |  |
| Huaier 3-6 m | 1.30(0.49–3.44) | 0.600 |  |  | 1.21(0.46–3.21) | 0.704 |  |
| Model 1: Huaier adjusted for Gender, AFP grade, TBIL grade, size grade, and ablation route.  Model 2: Huaier adjusted for Age, Hepatic virus, and ALB grade. | | | | | | | |

Abbreviation: PFS, Progression-free survival; OS, overall survival; AFP, alpha-fetoprotein; TBIL, total bilirubin; ALB, albumin.
