# Supplementary material for: Long-term oral administration of Huaier granules improves survival outcomes in hepatocellular carcinoma patients within Milan criteria following microwave ablation: a propensity score matching and stabilized inverse probability weighting analysis
Source: Front Pharmacol. 2024 May 7;15:1336347. doi: 10.3389/fphar.2024.1336347 (PMC11106438; doi:10.3389/fphar.2024.1336347)

**Supplementary File 2
“Long-Term Oral Administration of Huaier Granules Improves Survival Outcomes in Hepatocellular Carcinoma Patients Within Milan Criteria following Microwave Ablation: A Propensity Score Matching and Stabilized Inverse Probability Weighting Analysis”**

**PSM and Stabilized IPTW**

library(survival)

library(survminer)

library(openxlsx)

library(rms)

library(RISCA)

library(tableone)

library(survey)

library(MatchIt)

Total$Hepatopathy<-factor(Total$Hepatopathy)

#PSM

m.out<-matchit(data =Total,

formula =Huaier ~ Sex+Age+BMI+AFPgrade+Hepatopathy+PLT+ALT+AST+

Child+Cirrhosis+Hypersplenism+Multiple+Size+Ablation,

method = "nearest",

distance = "logit",

replace = FALSE,

caliper = 0.2,

ratio = 1)

summary(m.out)

A total 92 pairs of patients are matched, as detailed in shown.


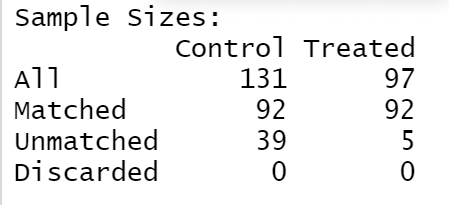


Export file

PSMMWA<-match.data(m.out)

write.xlsx(PSMMWA,

file = "PSM",

append = FALSE)

Survival analysis:

ipw.log.rank(times= PSMMWA$PFS,failures= PSMMWA $Relapse,variable= PSMMWA $Huaier)

ipw.log.rank(times= PSMMWA $OS,failures= PSMMWA $Death,variable= PSMMWA $Huaier)

ipw.log.rank(times= PSMMWA $EMS,failures= PSMMWA$EM,variable= PSMMWA $Huaier)


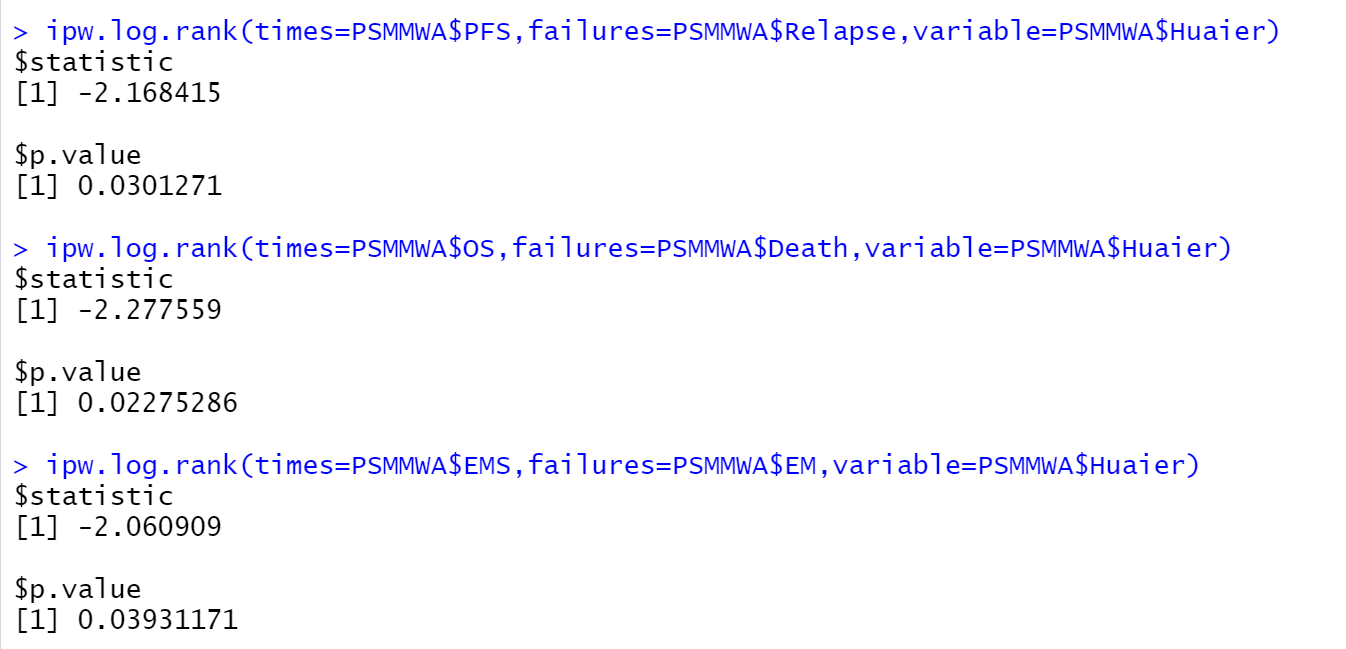


myVars<-c("Age","Agegrade","Sex","BMI","BMIgrade","AFPgrade","Hepatopathy",

"Child","Cirrhosis","Hypertension","ALB","BILT","ALT","AST",

"PLT","PT","Ablation","Size","Multiple")

catVars<-c("Agegrade","Sex","BMIgrade","AFPgrade","Hepatopathy",

"Child","Cirrhosis","Hypertension","Ablation","Multiple")

nonvar<-c("BMI","ALB","BILT","ALT","AST","PLT","PT","Szie")

dataPSM<-svydesign(ids=~1,data=Total,weights=~W)

tab_PSM_MWA=svyCreateTableOne(vars = myVars,strata = "Huaier",

data = dataIPTW,factorVars = catVars)

print(tab_PSM_MWA,showAllLevels=TRUE,smd=TRUE,nonnormal = nonvar)


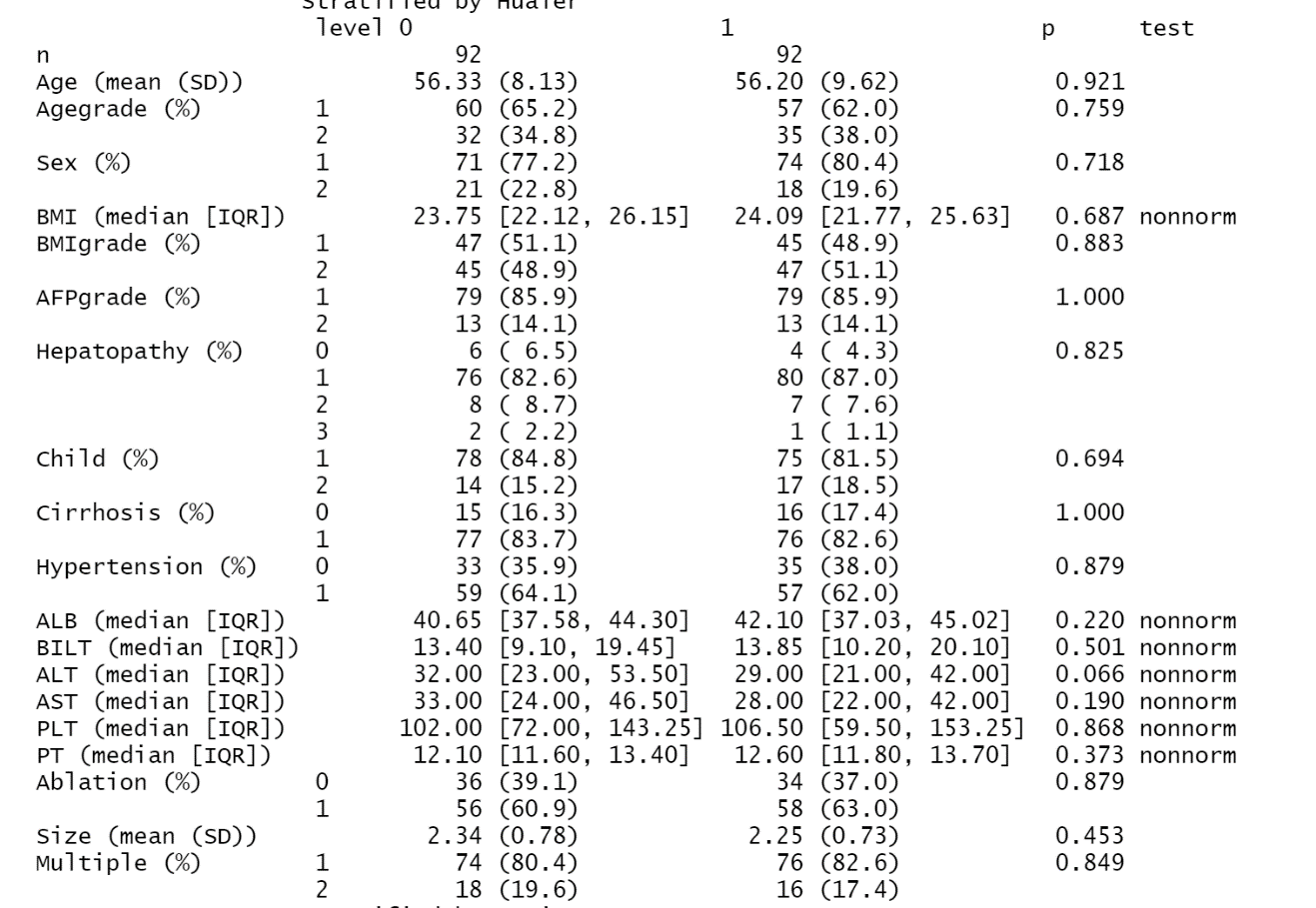


#Stabilized IPTW

Pr0 <- glm(Huaier~ 1,family = binomial(link="logit") ,data=Total)$fitted.values[1]

Pr1 <- glm(Huaier ~ Huaier ~ Sex+Age+BMI+AFPgrade+Hepatopathy+PLT+ALT+AST+

Child+Cirrhosis+Hypersplenism+Multiple+Size, data= Total,

family=binomial(link = "logit"))$fitted.values

pt<-0.4254

W<- (Total$Huaier==1) * (pt/Pr1) + (Total$Huaier==0) * (1-pt)/(1-Pr1)

Survival analysis:

ipw.log.rank(times=Total$PFS,failures=Total$Relapse,variable=Total$Huaier,W)

ipw.log.rank(times=Total$OS,failures=Total$Death,variable=Total$Huaier,W)

ipw.log.rank(times=Total$EMS,failures=Total$EM,variable=Total$Huaier,W)


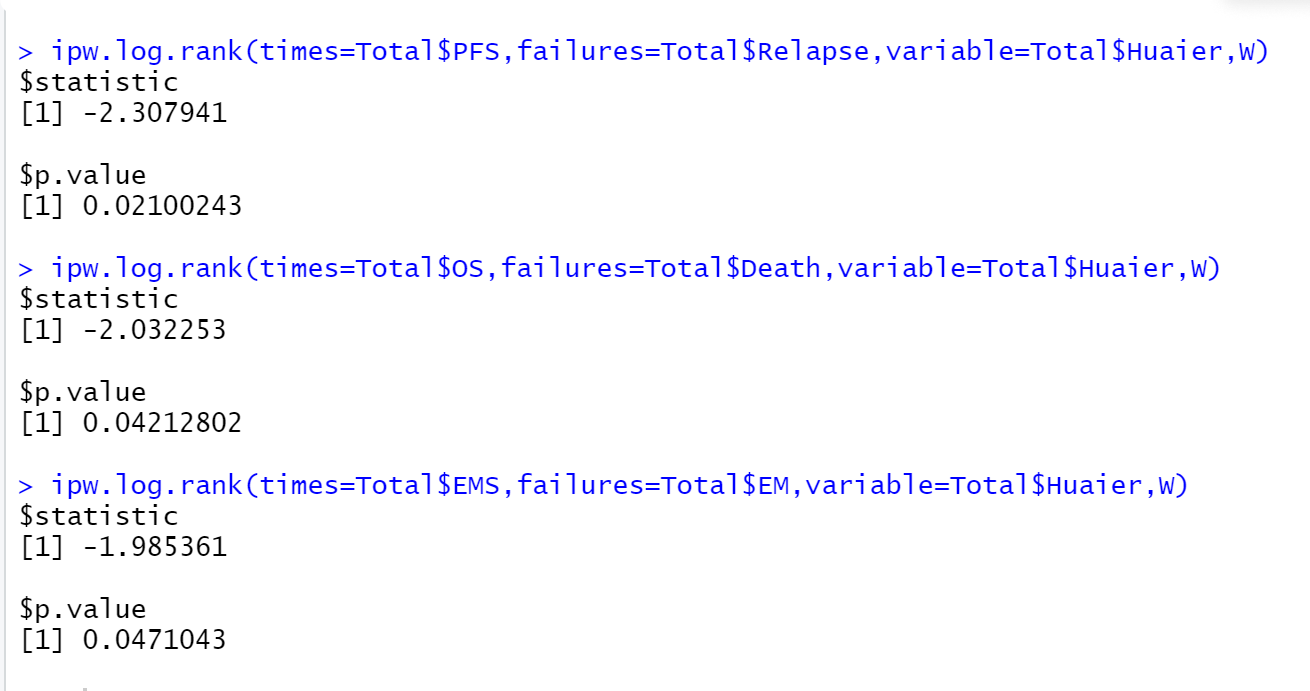


Baseline

myVars<-c("Age","Agegrade","Sex","BMI","BMIgrade","AFPgrade","Hepatopathy",

"Child","Cirrhosis","Hypertension","ALB","BILT","ALT","AST",

"PLT","PT","Ablation","Size","Multiple")

catVars<-c("Agegrade","Sex","BMIgrade","AFPgrade","Hepatopathy",

"Child","Cirrhosis","Hypertension","Ablation","Multiple")

nonvar<-c("BMI","ALB","BILT","ALT","AST","PLT","PT","Szie")

dataIPTW<-svydesign(ids=~1,data=Total,weights=~W)

tab_IPTW_MWA=svyCreateTableOne(vars = myVars,strata = "Huaier",

data = dataIPTW,factorVars = catVars)

print(tab_IPTW_MWA,showAllLevels=TRUE,smd=TRUE,nonnormal = nonvar)


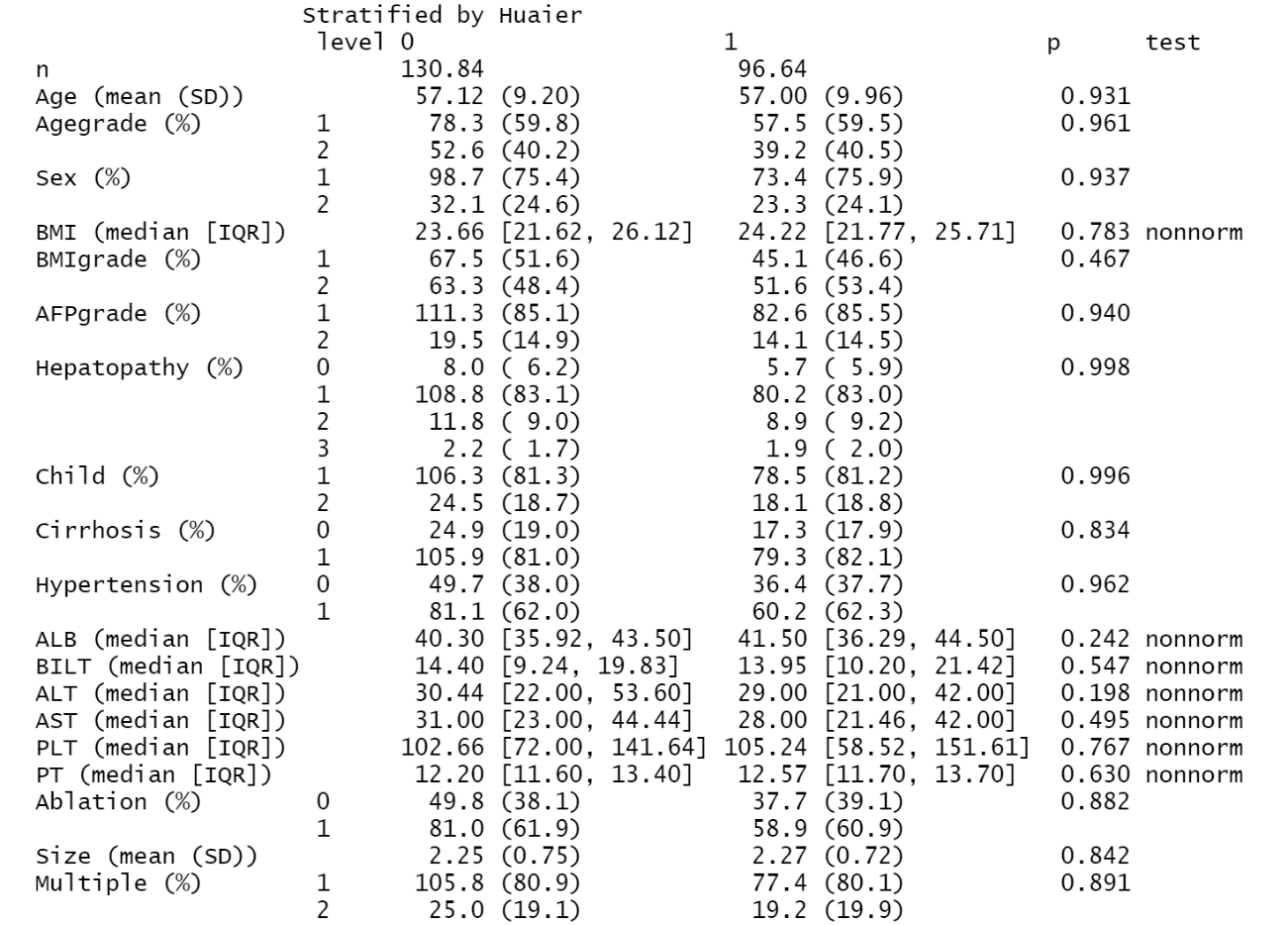

Supplement: Supplementary file 3 [file DataSheet2.docx]
