# Supplementary material for: Long-term oral administration of Huaier granules improves survival outcomes in hepatocellular carcinoma patients within Milan criteria following microwave ablation: a propensity score matching and stabilized inverse probability weighting analysis
Source: Front Pharmacol. 2024 May 7;15:1336347. doi: 10.3389/fphar.2024.1336347 (PMC11106438; doi:10.3389/fphar.2024.1336347)
Supplement: Supplementary file 4 [file DataSheet1.docx]

**Supplementary File 1
“Long-Term Oral Administration of Huaier Granules Improves Survival Outcomes in Hepatocellular Carcinoma Patients Within Milan Criteria following Microwave Ablation: A Propensity Score Matching and Stabilized Inverse Probability Weighting Analysis”**

1. **Manufacturer and GPS coordinates:** Qidong Gaitianli Pharmaceutical, Jiangsu, China. GPS coordinates: 121.62°E 31.82°N
2. **Source and proportion of** **plant extracts**：*Poriarobiniophila* (Murrill) Ginns (formerly named Trametesrobinioiophila Murr) is a sandy beige mushroom that was founded on the trunks of trees, having a nearly 1600 years of history in ancient China. The mian components of plant extract were proteoglycan, in which the polysaccharide content was 41.5%, the amino acid content was 12.93%, and the water content was 8.72%. Sucrose, dextrin and soluble starch were added at the ratio of 2:2:1 to form granule adjuvant. In the production of Huaier granules, chromatographic fingerprint analysis is used to illustrate the "plant equivalence" of the product for the purposes of raw material preparation and quality control, and to ensure that the ingredients meet the requirements of the State Food and Drug Administration of China (SFDA). (**Figure S1-3, Table S4**)
3. **Drug certification:** During March 1993 to June 1994, the phase III clinical trial about huaier Granules were performed (production certificate No. Z20000109). The first new drug certificate issued of huaier granules was obtained by the State Food and Drug Administration in 2002. Each lot is manufactured in accordance with Good Manufacturing Practice standards and complies with SFDA required quality assurance standards (Identification code: WS3-215(Z-029)-2001(Z)-2012; ybz042020032009z -2012) (**Figure S3-5, Table S1-3**)
4. **Description of material storage location and method:**

Location: 88 Heping South Road, Qidong Economic and Technological Development Zone, 1166 Nanyuan West Road, Huilong Town, Qidong, Jiangsu Province, China.

Method: Herbology special seal, avoid light, moistureproof storehouse.

**Figure S1 Fingerprint of *Poriarobiniophila* (Huaier)**


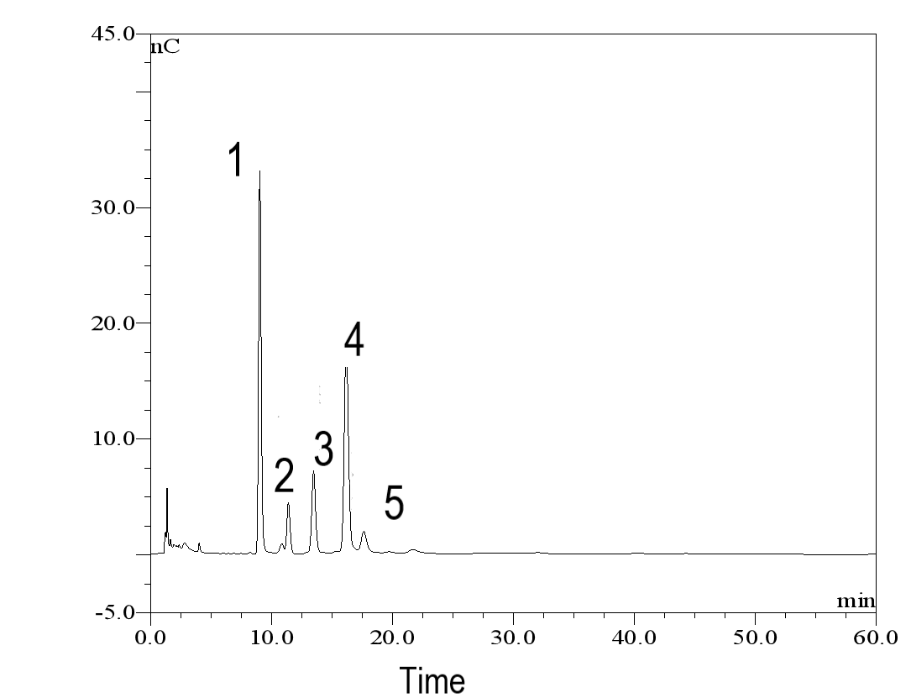


Note: 1. Arabinose 2. Galactose 3. Glucose 4. Xylose 5. Mannose

**Figure S2 Fingerprint of Huaier granules**


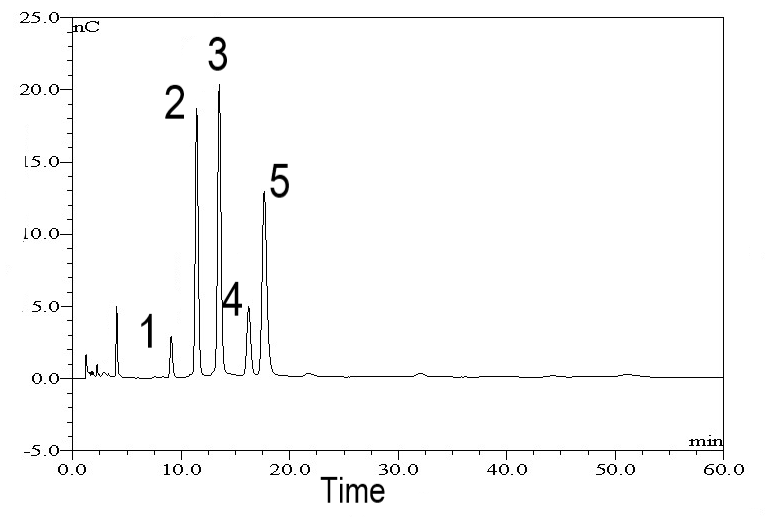


Note: 1. Arabinose 2. Galactose 3. Glucose 4. Xylose 5. Mannose . The chromatogram height of peak 3 is higher and peak 2 and peak 5 is lower.

**Table S1**

| Substances | Constitutions |
| --- | --- |
| Huaier aqueous extract | 41.5% carbohydrates+12.93% amino acids+8.72% water  Amino acids: Asp, Thr, Ser, Glu, Pro, Gly, Ala, Cys, Val, Met, Ile, Leu, Tyr, Phe, Lys, His, Trp, Arg  Monosaccharides: fucose, arabinose, xylose, mannose, galactose, glucose |

**Table S2**

**The State Food and Drug Administration**

**The Promulgated Approval of National Drug Standards（revision）**

The Certificate No. ZGB2012-18

| Drug Name | Chinese name： 槐耳颗粒  Chinese pinyin： Huaier Keli | | | | |
| --- | --- | --- | --- | --- | --- |
| Dosage Form | Granule | Applicant Company | | Qidong Gaitianli Pharmaceutical Co. Ltd | |
| Current Standards | Bureau standard | Review Organization | | Jiangsu Institute for Food and Drug Control | |
| Original Standard No. | YBZ04202003-2009Z | Authorized Organization | | Chinese Pharmacopoeia Commission | |
| Revised content and conclusion | According to the drug administration law and relevant regulations, after examination, in the "prescription", the "trametes robiniophila murrill（Huaier）" is changed to"Huaier extract" materials extracted from trametes robiniophila murrill；add "fingerprint" item；add the quality standard of Huaier extract in the notes. | | | | |
| Implementing regulations | Within six months from the date of promulgation of this standard, the drugs produced by the manufacturer according to the original standard shall still be tested according to the original standard. Drugs manufactured in accordance with this standard shall be tested in accordance with this standard. From the date of implementation of this standard, the manufacturer must produce the drug in accordance with this standard and test it in accordance with this standard. At the same time, the original standard shall not be used. | | | | |
| Standard No. | YBZ04202003-2009Z-2012 | | Implementation Date | | Apr. 30^th^ 2013 |
| Appendix | The pharmaceutical standard of Huaier granule | | | | |
| Send to | Food and Drug Administration of all provinces, autonomous regions and municipalities directly under the central government. Drug administration of Ministry of health of the PLA General Logistics Department, relevant manufacturers. | | | | |
| Copy to | Drug inspection institutes of all provinces, autonomous regions and municipalities directly under the central government, and drug and instrument inspection institutes of the Ministry of health of the PLA General Logistics Department. National Institutes for Food and Drug Control. National Committee for the protection and evaluation of traditional Chinese medicine varieties, drug evaluation center, certification center, evaluation center and information center. Department of drug safety supervision. Inspection Bureau of the SFDA. | | | | |

The State Food and Drug Administration

Oct. 30^th^ 2012

**Table S3**

| CERTIFICATE OF GOOD MANUFACTURING PRACTICES FOR PHARMACEUTICAL PRODUCTS PEOPLE’S REPUBLIC OF CHINA |
| --- |
| Certificate number: JS201808857 |
| Manufacturer: Qidong Gaitianli Medicine Co. Lid |
| Address:  88 Heping South Road, Qidong Economic and Technological Development Zone,  1166 Nanyuan West Road, Huilong Town, Qidong, Jiangsu Province, China. |
| Scope of Inspection: Bulk Drug (Huaier Junzhi): Granules, Pre-treatment and extraction of traditional Chinese medicine |
| This is to certify that the above-mentioned manufacturer complies with the requirements of Chinese Good Manufacturing Practices for Pharmaceutical Products. |
| This certificate remains valid until 19/7/2023 |
| Issued By JIANGSU FOOD AND DRUG ADMINISTRATION |
| Date for Issuing 20/7/2018 |
| CHINA FOOF AND DRUG ADMINISTRATION |

**Table S4**

| Drug Manufacturing Certificate |
| --- |
| Manufacturer: Qidong Gaitianli Medicine Co. Lid  Certificate number: SU20160297 |
| Social Credit Code: 91320681608384436X  Classification code: AzDz |
| Registered address:  88 Heping South Road, Qidong Economic and Technological development Zone  Daily supervision and management organization:  JIANGSU FOOD AND DRUG ADMINISTRATION |
| Legal person: WuWei Xu Complaints hotline: 12315 |
| Person in charge of enterprise: WuWei Xu  Issued By JIANGSU FOOD AND DRUG ADMINISTRATION |
| Quality supervisor: JuLan Xu Issuer: ChunPing Zhang |
| Product Address:  88 Heping South Road, Qidong Economic and Technological Development Zone,  1166 Nanyuan West Road, Huilong Town, Qidong, Jiangsu Province, China. |
| This certificate remains valid until 06/12/2025 |

**Table S5** The pharmaceutical batch numbers for 2011-2019

| Year | 2011 | 2012 | 2013 | 2014 | 2015 | 2016 | 2017 | 2018 | 2019 |
| --- | --- | --- | --- | --- | --- | --- | --- | --- | --- |
| The Batch Number | AC10 | BD15 | CC24 | DC13 | EE20 | FD24 | GE09 | HE08 | JM19 |
